# Transcriptome Analysis of Gene Expression Profiles of *Tomato Yellow Leaf Curl Virus*-Infected Whiteflies over Different Viral Acquisition Access Periods

**DOI:** 10.3390/insects11050297

**Published:** 2020-05-11

**Authors:** Meng Li, Jing Zhao, Yun-Lin Su

**Affiliations:** 1 School of Food and Bioengineering, Zhengzhou University of Light Industry, Zhengzhou 450000, China; 2 Institute of Insect Sciences, Zhejiang University, Hangzhou 310058, China; swuzhj19940215@163.com; 3 Key Laboratory of South China Agricultural Plant Molecular Analysis and Genetic Improvement, South China Botanical Garden, Chinese Academy of Science, Guangzhou 510650, China; suyunlin@scbg.ac.cn

**Keywords:** TYLCV, whitefly, AAPs, DEGs, metabolism, signal transduction

## Abstract

*Tomato yellow leaf curl virus* (TYLCV), which is transmitted by *Bemisia tabaci* in a persistent-circulative manner, threatens tomato production worldwide. Little is known about the complicated interaction during this process at the molecular level. In this study, viral AAPs at 0 h, 2 h, 6 h, 12 h and 48 h were investigated using a comparative transcriptome analysis to uncover the transcriptional responses of whiteflies to virus infection. Our results have shown that 755, 587, 1140 and 1347 differentially expressed genes (DEGs) were identified in the comparisons of the data of 0 h vs. 2 h, 0 h vs. 6 h, 0 h vs. 12 h and 0 h vs. 48 h, respectively. KEGG analysis showed that DEGs associated with metabolisms and signal transduction were down-regulated in virus-infected whiteflies. Additionally, 16 up-regulated putative transporter genes and 10 down-regulated genes associated with IL-17 signaling pathway were identified by time-associated gene cluster analysis. These data boost our comprehensions on whitefly-TYLCV interactions associated with different viral AAPs.

## 1. Introduction

Plant virus diseases threaten crop yield and cause tremendous losses of agricultural crops worldwide [1,2,3]. In general, plant viruses are transmitted by insect vectors in four manners as follows: non-persistent, semi-persistent, persistent-circulative and persistent-propagative [4,5,6]. Begomoviruses (genus *Begomovirus*, family *Geminiviridae*) are transmitted in a persistent-circulative manner by the whitefly *Bemisia tabaci* [7,8,9,10]. They are a group of destructive plant viruses that affect many important crops such as tomato [2] and cassava [11].

*Tomato yellow leaf curl virus* (TYLCV) is one of the most devastating begomoviruses in the ‘top 10’ plant viruses list [2]. Among the known 39 cryptic species of *B. tabaci* [12,13,14], as far as we know, there are only two invasive species, Middle East Asia Minor 1 (MEAM1) and Mediterranean (MED), that acquire and transmit TYLCV with high efficiency [15]. After being ingested by whiteflies, TYLCV virions can travel from the midgut lumen into the haemolymph, then move to the salivary gland with the flow of haemolymph and finally be inoculated into healthy plants with saliva excretion [5]. In this process, numerous genes are regulated in the whiteflies. Li et al. [16] and Luan et al. [17] suggested that acquisition of *Tomato yellow leaf curl China virus* (TYLCCNV) influenced the expression of many genes related to metabolism, cell cycle and immune reactions in whiteflies. Hasegawa et al. [18] and Geng et al. [19] also demonstrated that the infection of TYLCV induced transcriptional changes of the genes involved in cell cycle and antiviral immunity. Furthermore, expression of the genes of receptors, trafficking proteins, cuticle proteins and defense related proteins could also be interfered with by begomoviruses in vectors [18,19,20]. 

Previous study indicated that TYLCV could accomplish the circulation from midgut to salivary gland within 8 h [21]. Furthermore, viruses could be detected in midgut, haemolymph and salivary glands in whiteflies after an acquisition access period (AAP) of 1 h, 2 h and 7 h, respectively [21,22]. After a viral AAP of 12–48 h, the amount of viruses in whiteflies reached a very high level of abundance [23]. Gene expression of whiteflies were extensively studied to unravel the mechanisms of whitefly-virus interaction. However, as far as we know, those studies only conducted the comparisons of gene expression profiles between the whiteflies feeding on healthy plants and those feeding on virus-infected plants over 24 h [16,17,18,19,20]. Therefore, expression patterns of whitefly genes in the early stage of virus-vector interaction and the temporal regulation in whiteflies with the gradual accumulation of virus in vectors remains unclear. In this study, viral AAPs at 0 h, 2 h, 6 h, 12 h and 48 h were used for comparative transcriptome analysis to further explore the mechanisms in virus-vector interaction. Our work sought to answer the following two questions: (1) regardless of viral amounts, does gene regulation change in TYLCV-infected whiteflies; (2) does any temporally up-regulated or down-regulated genes or pathways exist in TYLCV-infected whiteflies? These answers may help to reveal the begomeovirus-whitefly interactions, especially in the early stage, and to identify novel vector factors in the transmission of TYLCV.

## 2. Materials and Methods 

### 2.1. Whitefly Samples for RNA-Seq

MEAM1 whitefly colony (mtCOI GneBank Accession code: GQ332577) was maintained on cotton plants (*Gossypium hirsutum* L. cv. Zhemian 1793) in insect proof cages. TYLCV-infected tomato (*Solanum lycopersicum* Mill. cv. Hezuo903) plants were obtained using the procedures as described before [15]. Five- to seven-day-old MEAM1 adults were collected and transferred to TYLCV-infected tomato plants for different periods. Insect rearing, plant cultivation and sample collection were conducted at 26 ± 1 °C, a 14:10 photo: scoto period and a relative humidity of 60 ± 10%.

To determine the acquisition efficiency and virus titers in whiteflies after different AAPs on TYLCV-infected plants, approximately 500 whitefly adults were released onto TYLCV-infected tomato plants for 1 h, 2 h, 6 h, 8 h, 12 h, 24 h and 48 h. Following each AAP, 23–24 female adults were collected and assayed individually for the detection of TYLCV DNA by PCR as described in [15]. Whiteflies with an AAP of 2 h, 6 h, 12 h and 48 h were subjected to DNA extraction for quantitative RT-PCR (qPCR) analysis, as described previously [24]. The primers used in PCR/qPCR analyses were listed in Table 1. 

As to RNA-seq analysis, each AAP (2 h, 6 h, 12 h and 48 h) consisted of three biological replicates and each biological replicate included 200 whitefly adults. Whiteflies with no access to virus-infected plants were used as control (0 h). In total, 15 samples (3 replicates × 5 AAPs) were collected for transcriptome sequencing.

### 2.2. RNA Isolation and cDNA Library Constructions for RNA-Seq

Total RNA of each sample was extracted using Trizol (Invitrogen, Carlsbad, CA, USA) according to the manual instructions. Genomic DNA contamination was removed using RNase-free DNaseI (Takara, Kusatsu, Shiga, Japan). The quantity and quality of total RNA were evaluated by Nano Drop (Thermo Fisher Scientific, Waltham, MA, USA) and Agilent 2100 bioanalyzer (Agilent Technologies, Santa Clara, CA, USA), respectively. When the RNA integrity number (RIN) of a sample was greater than six, 1000 ng of total RNA was used as input. The total RNA was firstly treated by Oligo (dT)-attached magnetic beads for mRNA purification [25]. Then, cDNA library was constructed as follows: (1) fragmenting mRNA into small piece; (2) generating first-strand cDNA with random hexamer-primed reverse transcription, followed by a second-strand cDNA synthesis; (3) adding A-Tailing Mix AND RNA Index adapters to end repair by incubation; (4) amplifying the cDNA fragments obtained from last steps by PCR, purifying PCR products by Ampure XP Beads, and dissolving the PCR products in EB solution; (5) validating products on the Agilent Technologies 2100 bioanalyzer for quality control; (6) denaturing the double strand PCR products from step (5) by heating and circularizing by the splint oligo sequence to produce the final library; (7) amplifying the final library with phi29 to make DNA nanoball (DNB), which had more than 300 copies of one molecule.

### 2.3. Transcriptiome Sequencing and Clean Reads Mapping

DNBs were loaded into nanoarray and then sequenced for 100 bp paired-end reads on BGISEQ-500 platform (BGI-Shenzhen, China). The raw reads generated from sequencing were cleaned by removing adaptor-polluted reads, reads with unknown sequences “N” accounting for more than 5%, and low-quality reads (more than 20% bases with Phred threshold score < 10). The clean reads were mapped to the MEAM1 whitefly genome (http://www.whiteflygenomics.org) using the software of Hierarchical indexing for spliced alignment of transcripts (HISAT) v2.1.0 [26].

### 2.4. DEGs Analysis

Reads per kilobase million mapped reads (RPKM) was used to estimate gene expression levels. It could eliminate the bias caused by effects of sequencing depth and gene length. Fold changes of gene expressions were analyzed by the software of DEGseq [27] and differentially expressed genes (DEGs) were identified with the threshold of Q-value (adjusted *p*-value) ≤0.001 and log2 (fold change) ≥1. 

### 2.5. GO and KEGG Analysis

Gene ontology (GO) and Kyoto encyclopedia of genes and genomes (KEGG) functional classifications were performed using Blast2GO and KOBAS (http://kobas.cbi.pku.edu.cn). KEGG enrichment analysis was conducted by using R phyer. *p*-value was corrected by false discovery rate (FDR). The pathways with FDR <0.01 was considered to be significantly enriched. Data mining and figure presentation process were analyzed by BGI in-house customized data mining system called Dr.Tom (http://biosys.bgi.com).

### 2.6. qPCR Validation

Ten DEGs were selected for qPCR analysis to validate the data obtained by RNA-seq. Thirty whitefly adults were collected as one sample at each AAP. Three replicates were performed. Total RNA was extracted using Trizol (Invitrogen, Carlsbad, CA, USA) following the manual protocol. Reverse transcription was conducted by using PrimeScript RT reagent Kit (TaKaRa, Kusatsu, Shiga, Japan). The qPCR was performed on the CFX96^TM^ Real-Time PCR Detection System (Bio-Rad, Hercules, CA, USA) with SYBR Premix Ex ExTaq II (Takara, Kusatsu, Shiga, Japan). We used β-actin as a reference gene. Relative expression level of DEGs was calculated by 2^−ΔCt^. Primers used in this experiment were listed in Table 1.

## 3. Results

### 3.1. Sequencing and Annotation of Transcriptomes

**Figure 1:**
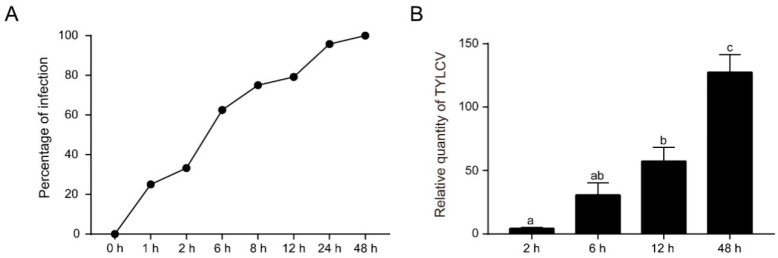
*Tomato yellow leaf curl virus* (TYLCV) accumulates over viral acquisition periods in whiteflies. (**A**) The percentage of viral infection increases with viral acquisition access periods. (**B**) The quantity of viruses in whiteflies continually accumulate over viral feeding periods. MEAM1 adults post emergence 5–7 d were released on TYLCV-infected tomato plants. At each time point, 23–24 individuals were collected for virus detection and quantification. ANOVA-LSD (L) test (*p* < 0.05) was performed with SPSS 20.0 (IBM, Armonk, NY, USA). *p* = 0.064 for 2 h vs. 6 h, *p* = 0.064 for 6 h vs. 12 h, and *p* < 0.001 for other comparisons.

As shown in Figure 1, the percentage of the whiteflies infected with TYLCV increased with the AAP up to 48 h. Additionally, the raised viruses quantity in whiteflies paralleled the increasing viral feeding time. To investigate the gene expression patterns, the whiteflies after an AAP of 0 h, 2 h, 6 h, 12 h and 48 h were subjected to RNA-seq analysis. The relevant data were deposited in the SRA as BioProject PRJNA562795. Fifteen cDNA libraries were constructed and ~21 M clean reads per library were obtained from the transcriptome sequencing (Table S1). On average, 91.81% and 77.95% of these reads were mapped and uniquely mapped to the MEAM1 whitefly genome, (http://www.whiteflygenomics.org), respectively. Furthermore, the reads were mapped to 15,662 genes of the whitefly genome. Pearson’s correlation coefficients indicated the high reproduce of the data in different replications (Figure S1).

### 3.2. DEGs in Virus-Infected Whiteflies

**Figure 2:**
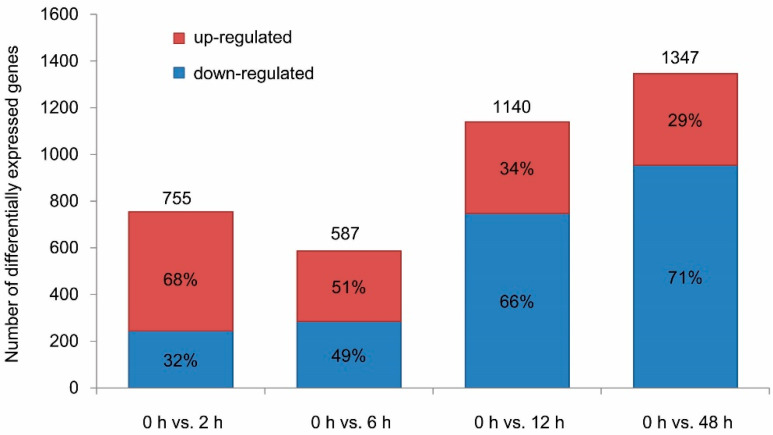
Differentially expressed genes (DEGs) in virus-infected whiteflies.

To reveal the regulation of gene expression upon acquisition of TYLCV, we compared the data in four ways, 0 h vs. 2 h, 0 h vs. 6 h, 0 h vs. 12 h and 0 h vs. 48 h. Herein, 755, 587, 1140 and 1347 DEGs were identified in the comparisons of the data of 0 h vs. 2 h, 0 h vs. 6 h, 0 h vs. 12 h and 0 h vs. 48 h, respectively (Figure 2), indicating that the numbers of DEGs tend to increase with the duration of AAPs (Figure 2). Among the DEGs, the percentage of down-regulated genes increased, while the up-regulated genes decreased throughout the entire AAPs (Figure 2). This suggested that TYLCV had significant influence on the gene expression of its vector, and, moreover, the effect became stronger as the virus amount increased in the whitefly population. 

### 3.3. Common DEGs in Virus–Infected Whiteflies Indicated the Perturbance of Vector Metabolisms and Signal Transductions by Tylcv Infection

**Figure 3:**
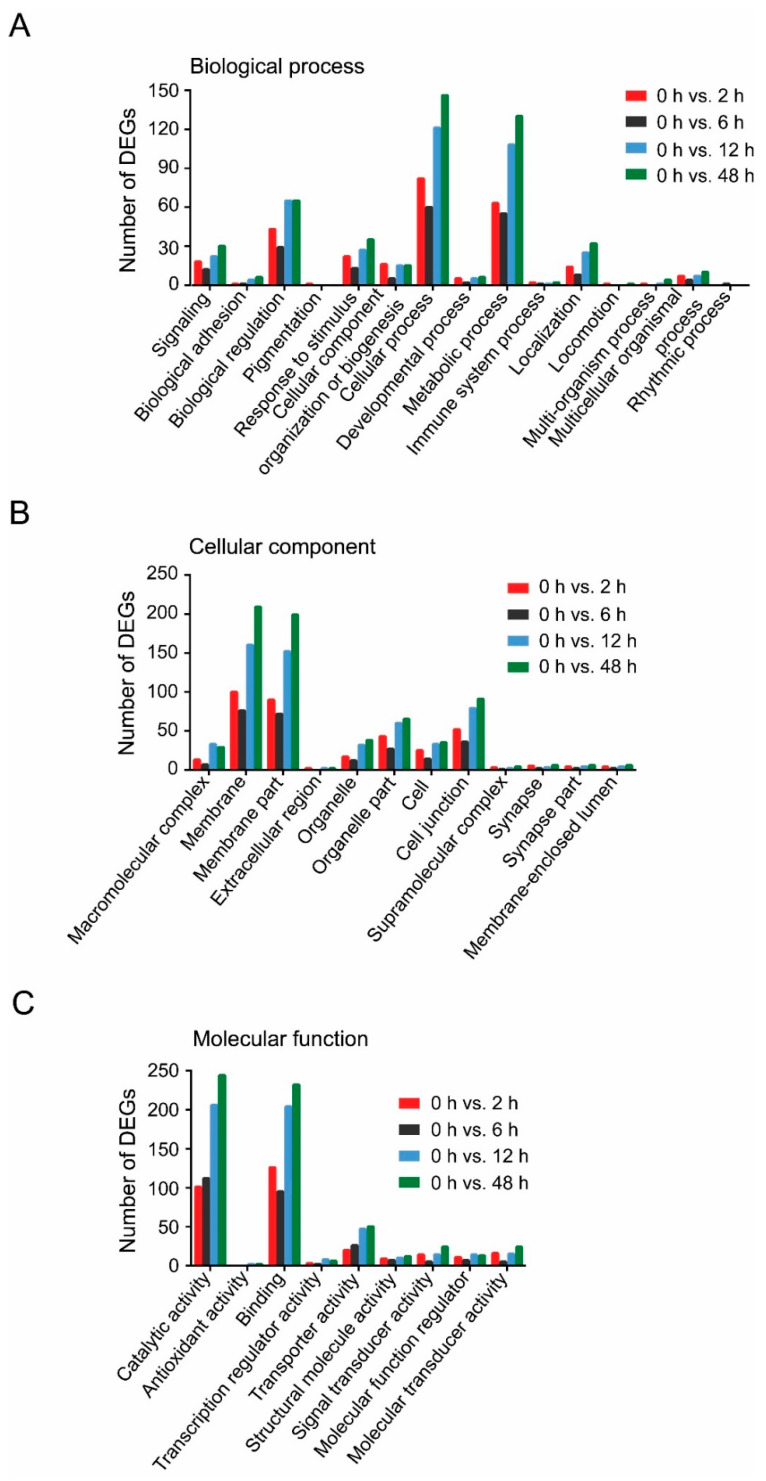
GO categories of DEGs. (**A**) Biological process; (**B**) Cellular component; (**C**) Molecular function. Different colors represent different comparisons.

GO assignments were performed to reveal the putative functions of DEGs. DEGs were categorized into 38 secondary GO categories in three biological concepts including biological process, cellular component and molecular function (Figure 3). Cellular process, membrane and catalytic activity were the most presented categories in biological process, cellular component and molecular function, respectively (Figure 3). The analysis of GO categories suggested that the DEGs in TYLCV-infected whiteflies at different viral AAPs had similar functional classifications in the biological process, cellular component and molecular function (Figure 3).

**Figure 4:**
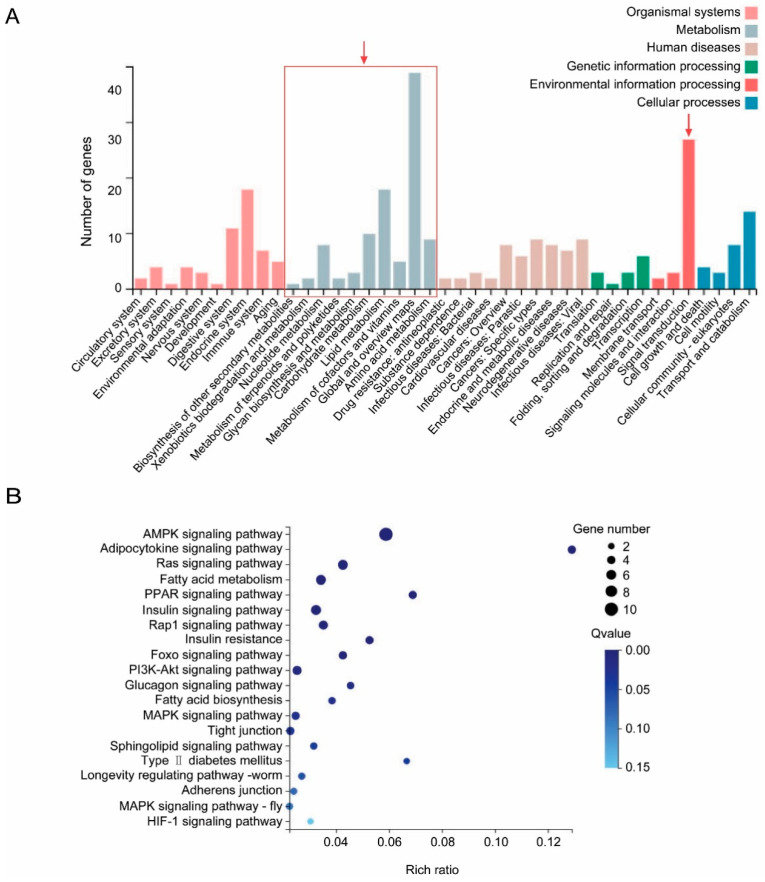
KEGG classifications and enrichment analysis of 235 common DEGs regardless of viral feeding time. (**A**) KEGG classifications, different colors stand for different functions. (**B**) KEGG enrichment analysis of DEGs related to signal transduction. The greater rich ratio and lower Q value mean greater intensiveness.

A total of 235 common DEGs were found among different comparisons (Figure S2). Hierarchical cluster analysis generated by pheatmap R indicated a consistent up- or down-regulation of these common DEGs among different comparisons (Figure S2). The result of the KEGG analysis suggested that 118 common down-regulated DEGs were related to metabolisms, signal transduction, organismal systems, genetic or environmental information processing, cellular process and human diseases (Figure 4). Among the down-regulated DGEs, a large proportion of them were classified in metabolism groups, especially for lipid metabolism, carbohydrate metabolism and amino acid (Figure 4). As for the category of signal transduction, 27 common down-regulated DEGs were found to be implicated in 13 signaling pathways such as AMPK signaling pathway, ras signaling pathwaysig, insulin signaling pathway and others (Figure 4, Table S2). 

### 3.4. Time-Associated Gene Cluster Analysis Suggested Activated Transporters But Impaired Il-17 Signaling Pathway in Virus-Infected Whiteflies

**Figure 5:**
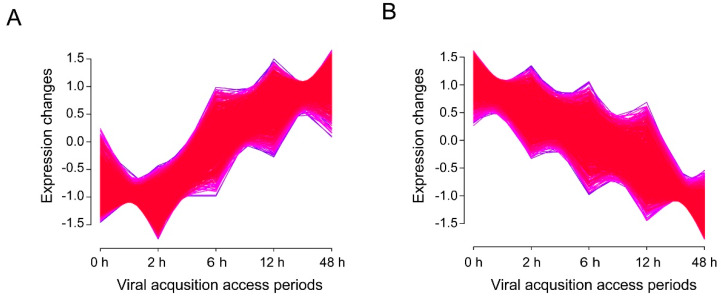
Time-associated gene clusters. For different treatments, genes with similar expression patterns were clustered into different groups. (**A**) Expression pattern of 1168 continually up-regulated genes. (**B**) Expression pattern of 1312 continually down-regulated genes.

To examine the temporal shift in gene expression during viral acquisition, time-associated gene cluster of all 15,662 genes was conducted by Mfuzz v2.34.0 [25,28]. It was notable that 1168 genes were found to be continually up-regulated (Figure 5A) and 16 genes encoding transporters were identified (Table 2). In addition, 1312 genes were continually down-regulated (Figure 5B) and 10 genes associated with interleukin (IL)-17 signaling pathway were identified (Table 3). Hierarchical cluster analysis of 127 up-regulated DEGs and 355 down-regulated DEGs in the comparison of 0 h vs. 48 h was shown in Figure S3.

### 3.5. qPCR Validation of DEGs Identified by RNA-Seq

**Figure 6:**
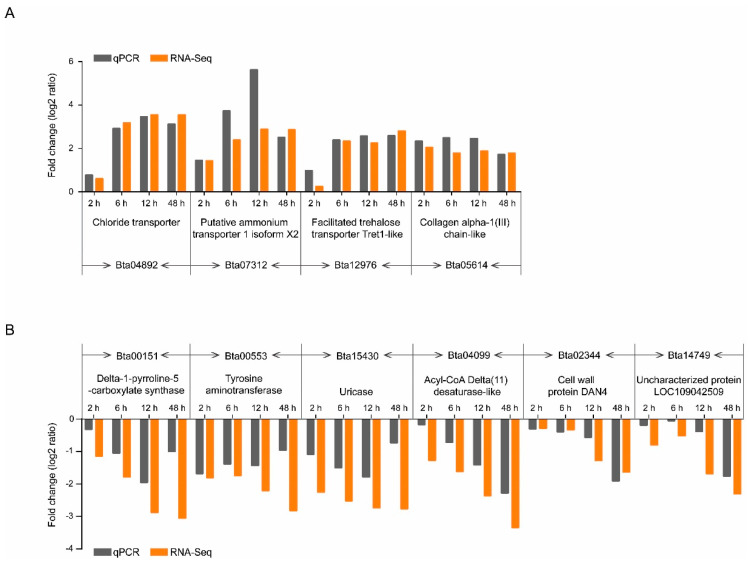
Expression patterns generated by the results of RNA-seq and RT-qPCR analyses. Four up-regulated and six down-regulated DEGs identified by RNA-seq were validated by comparative CT (ΔCT) RT-qPCR with β-actin as the control.

Expression levels of 10 DEGs, i.e., 4 up-regulated DEGs (Figure 6A) and 6 down-regulated DEGs (Figure 6B), were verified by qPCR. The results showed that no significant difference in the trends of gene regulation compared to the results generated from RNA-seq analysis, suggesting that the transcriptome data were reliable in this study.

## 4. Discussion

Several studies had analyzed the gene expression profiles of TYLCV-infected whiteflies (with the AAPs longer than 24 h) using high-throughput RNA-sequencing and comparative transcriptome analysis [17,18,19,20]. To reveal more details in the early-stage interactions between whiteflies and TYLCV, gene expression profiles of TYLCV-infected whiteflies with the AAPs less than 12 h were investigated in this study. Our results indicated that hundreds of genes were up- or down-regulated as a consequence of viral infection. Numbers of DEGs tend to increase with the duration of AAPs, which may be due to the accumulation of TYLCV in whitefly populations. Interestingly, as the percentage of down-regulated genes increased, while the up-regulated genes decreased during the extending AAPs, we deduced that the repression of vector genes might facilitate the infection of TYLCV in *B. tabaci*. It should be pointed out that, in addition to the response to virus infection, the changes in gene expressions of whiteflies may also be caused by the adaptation to new hosts [29]. In particular, we noticed that the number of DEGs identified in the comparison of 0 h vs. 2 h (755) is higher than that of 0 h vs. 6 h (587). The number of DEGs identified by comparing expression profiles of whiteflies with different AAPs with that of control (0 h) may be higher than the actual.

As was previously observed in [18], our results showed that the genes clustered in the groups of the metabolisms involving lipid, carbohydrate and amino acid metabolism were down-regulated in virus-infected whiteflies. Similarly, disturbances of amino acid metabolisms in whitefly were found to be associated with TYLCCNV infection [17] and manipulations of metabolisms by plant viruses were reported in other insect vectors [30]. TYLCV infection of MEAM1 reduced the whitefly’s fecundity and longevity [31]. Longevity of MED was also found to be shortened by TYLCV infection [32]. These deleterious effects of TYLCV could be caused by the dramatic repression of the genes involved in basic metabolisms including lipid, carbohydrate and amino acid metabolisms.

On the other hand, interference of signaling pathway is a universal phenomenon in vector-virus interactions [18,33]. Herein, 13 signaling pathways were predicted to be down-regulated in TYLCV-infected whiteflies. Genes involved in the 20 signaling pathway were also found to be down-regulated in *Tomato chlorosis virus*-infected whiteflies [33]. Among the down-regulated pathways, AMPK and PI3K-Akt signaling pathways are associated with cellular apoptosis and autophagy [34,35,36,37,38,39]. The regulation of PI3K-Akt signaling pathway by animal viruses have been extensively studied in mammals [40]. Viruses may stimulate [41,42,43,44] or inhibit [45,46] PI3K-Akt signaling pathway to reduce or activate apoptosis in host cells to facilitate their infection. However, roles of pi3k-akt signaling pathway in the interactions between the viruses and whiteflies remain unknown.

Time-associated gene cluster analysis revealed the continuous down-regulation of the genes involved in IL-17 signaling pathway over the AAPs. IL-17 signaling has been shown to play critical role in host defense during bacterial infection [47]. However, as for virus, two studies reported that the IL-17 acted as a pathogenic factor in human [48,49]. The function of IL-17 signaling in viral infection to insect vector was unclear to date due to insufficient study. The suppression of IL-17 signaling pathway by TYLCV might assist the persistence of virus through the suppression of the down-stream immune reaction in the vector.

After the long-term coadaption and coevolution of begomoviruses and whiteflies, whiteflies have developed defenses systems to prevent virus invasion, and meanwhile, viruses have evolved the capacity of utilizing cellular proteins in whitefly to assist their transmission. Viral transportation plays an important role in virus transmission, and whitefly cellular proteins acts complicatedly in the interplays between whitefly and begomoviruses. Up to now, several whitefly proteins had been uncovered their favors in viral transport [50,51,52,53,54,55]. A midgut protein, cyclophilin B and collagen, has been characterized to act positively in viral transport [50,51,52]. On the contrary, heat shock protein 70, knottin-1 and vesicle associated membrane protein-associated protein B have been proved to cause the inhibition in viral transport [53,54,55]. Additionally, the increasing omics-data uncover more and more whitefly proteins involved in viral circulative transport. For example, a large number of receptors and transporters, including sugar transporter ERD6-like 6, proton-coupled amino acid transporter, facilitated trehalose transporter, genes with cargo receptor activity, ATP-binding cassette (ABC) transporters, cuticle proteins, laminin subunit alpha, dystroglycan and integrin alpha-PS2, were found to be associated with viral transport in whiteflies [18,19,20]. In this study, in addition to facilitated trehalose transporter, some uncharacterized proteins predicted to be with transporter activity were identified in virus-infected whiteflies. These genes might also be crucial in viral transport. The functions of these identified genes/proteins in viral transmission could be further characterized by RNAi. 

## 5. Conclusions

In summary, we uncovered the transcriptional responses of *B. tabaci* after feeding on TYLCV-infected plants for 2 h, 6 h, 12 h and 48 h. Our results indicated that hundreds of genes were up- or down-regulated as a consequence of viral infection. Genes involved in metabolisms and signaling transduction were down-regulated in virus-infected whiteflies. Furthermore, 16 up-regulated putative transporter genes and 10 down-regulated genes associated with IL-17 signaling pathway were identified by time-associated gene cluster analysis. This study provided new clues to unravel the mechanisms of whitefly-begomovirus interactions. 

## Figures and Tables

**Table 1:** Primers for PCR/qPCR analysis.

Primer	Sequence (5′-3′)	Product Size (bp)	Annealing Temperature (°C)
PCR TYLCV-detection-F	ATCGAAGCCCTGATGTTCCTCGTGG	683	55
TYLCV-detection-R	CAGAGCAGTTGATCATGTATTGTATG		
qPCR TYLCV-qPCR-F	GAAGCGACCAGGCGATATAA	192	60
TYLCV-qPCR-R	GGAACATCAGGGCTTCGATA		
β-Actin-qPCR-F	TCTTCCAGCCATCCTTCTTG	174	60
β-Actin-qPCR-R	CGGTGATTTCCTTCTGCATT		
Bta04892-F	TCGGCAAGCCCTACATCATC	145	60
Bta04892-R	GGGCAGACCTCTTCAACGAT		
Bta12976-F	CCGACCAATGTGAAGGGTCA	77	60
Bta12976-R	AGTTTGTTGGTGGCGAAGGA		
Bta12500-F	CAACCCTGGTCACGTATCCT	145	60
Bta12500-R	ACTCCTTCAAATTCCCCCGC		
Bta05614-F	CCGGCTTTTTCGACTATGCG	77	60
Bta05614-R	TGTCGGGGTCGAATCTGTTG		
Bta02344-F	ACGGGTGAAGACGAGAAACC	85	60
Bta02344-R	ACTGATTTTCCTCGCCCTCG		
Bta05747-F	GAGGCTGTCCCAGATCACAC	146	60
Bta05747-R	TTGGCCTTTGTAGCCACGAT		
Bta04099-F	CGAGCTGTGGGACTACAAGG	88	60
Bta04099-R	CACCGTCTTTCGCTCGTAGA		
Bta00151-F	AGGACATCAACGAGGCCAAG	83	60
Bta00151-R	ATTCTTCAGCTTGGACGGGG		
Bta00553-F	ATTGATCGCTCGAAGCACCT	81	60
Bta00553-R	AACTCCTTGCTGGGATTCGG		
Bta15430-F	ATTCCATCCGCGAGTGTGAG	81	60
Bta15430-R	TTCCATCGTTGTCACCCGTT		

**Table 2:** The expression profiles of the temporally up-regulated genes encoding transporters after different AAPs on TYLCV-infected plants.

Gene ID	Accession No.	Gene Annotation	log2 (Fold Change)			
			0 h vs. 2 h	0 h vs. 6 h	0 h vs. 12 h	0 h vs. 48 h
Bta00780	XP_021008519.1	gamma-aminobutyric acid receptor subunit epsilon	−0.90	2.94	3.72	4.28
Bta00927	PSN49542.1	V-type proton ATPase subunit H	-	3.63	4.75	4.48
Bta01800	XP_018900560.1	sodium-coupled monocarboxylate transporter 2-like	0.13	1.89	1.61	2.33
Bta04724	XP_018908139.1	solute carrier family 25 member 35-like	0.05	0.97	1.20	1.31
Bta04889	XP_023109180.1	vegetative cell wall protein gp1-like, partial	0.34	3.47	3.83	3.68
Bta04890	XP_010186100.1	uncharacterized protein DDB_G0271670-like, partial	0.65	2.80	3.04	3.02
Bta04892	KFH08892.1	chloride transporter, chloride channel (ClC) family protein	0.61	3.19	3.56	3.56
Bta04893	XP_023109180.1	vegetative cell wall protein gp1-like, partial	−0.29	3.76	4.12	4.05
Bta04894	XP_009899731.1	mucin-2	0.58	1.51	2.15	2.11
Bta04923	XP_018636297.1	chloride transporter, chloride channel (ClC) family protein	0.79	2.57	2.88	2.71
Bta04929	XP_018918095.1	uncharacterized protein LOC109044714	0.80	2.42	2.74	2.59
Bta07312	XP_018896664.1	putative ammonium transporter 1 isoform X2	1.44	2.39	2.89	2.87
Bta11245	XP_018900402.1	facilitated trehalose transporter Tret1-like isoform X2	0.36	0.89	1.25	1.25
Bta12976	XP_018901925.1	facilitated trehalose transporter Tret1-like	0.26	2.34	2.25	2.81
Bta14126	XP_011498176.1	zinc finger CCCH domain-containing protein 13-like	0.88	2.71	3.34	3.40
Bta15384	XP_018915842.1	zinc transporter ZIP3-like	1.49	2.96	2.80	2.86

**Table 3:** The expression profiles of the temporally down-regulated genes in the IL 17 signalling pathway after different AAPs on TYLCV-infected plants.

Gene ID	Accession No.	Gene Annotation	log2 (Fold Change)			
			0 h vs. 2 h	0 h vs. 6 h	0 h vs. 12 h	0 h vs. 48 h
Bta02344	XP_017068262.1	cell wall protein DAN4	−0.28	−0.33	−1.28	−1.63
Bta02727	XP_018907479.1	adhesive plaque matrix protein–like	−0.64	−0.40	−1.00	−1.26
Bta02853	XP_018908670.1	extracellular signal–regulated kinase 2	−0.34	−0.84	−0.68	−1.13
Bta06169	XP_018896164.1	uncharacterized protein LOC109029914	−0.30	−0.12	−0.47	−1.37
Bta07157	XP_018910204.1	uncharacterized protein LOC109039249	−0.18	−0.96	−2.37	−1.78
Bta07860	XP_018897127.1	uncharacterized protein LOC109030562 isoform X6	−0.49	−0.45	−0.89	−2.52
Bta08110	XP_018897436.1	uncharacterized protein LOC109030770	−0.26	−0.61	−0.92	−1.21
Bta11904	XP_018913143.1	uncharacterized protein LOC109041298	−0.53	−0.28	−1.16	−1.50
Bta12179	XP_018901010.1	D(2) dopamine receptor isoform X2	−0.94	−0.53	−1.15	−1.66
Bta14749	XP_018914842.1	uncharacterized protein LOC109042509	−0.80	−0.51	−1.68	−2.30

## Supplementary Materials

The following are available online at https://www.mdpi.com/2075-4450/11/5/297/s1, Figure S1: Pearson correlation coefficient of gene expression levels among 15 samples, Figure S2: Common DEGs in virus-infected whiteflies, (**A**) Venn-diagram showing unique and common DEGs in different comparisons. (**B**) Hierarchical cluster analysis of 235 identified common DEGs, Figure S3: Time-associated gene clusters. (**A**) Hierarchical cluster of 127 up-regulated genes. (**B**) Hierarchical cluster of 355 down-regulated genes, Table S1: Summary of RNA-Seq datasets generated from whiteflies fed on TYLCV-infected tomato plants for 0, 2, 6, 12 and 48 h, Table S2: Common DEGs involved in signal transduction in virus-infected whiteflies.
