# Supplementary material for: Transcriptome Analysis of Gene Expression Profiles of Tomato Yellow Leaf Curl Virus-Infected Whiteflies over Different Viral Acquisition Access Periods
Source: Insects. 2020 May 11;11(5):297. doi: 10.3390/insects11050297 (PMC7290374; doi:10.3390/insects11050297)
Supplement: Supplementary file 1 [file insects-11-00297-s001.pdf]

insects

Supplementary materials

Tables

Table S1. Summary of RNA-Seq datasets generated from whiteflies fed for 0, 2, 6, 12 or 48 h on TYLCV-infected tomato plants.

Sample	Total Raw Reads (M)	Total Clean Reads (M)	Total Mapping (%)	Uniquely Mapping (%)
0 h-1	21.46	21.41	92.04	78.86
0 h-2	21.47	21.41	92.10	77.55
0 h-3	21.46	21.32	92.03	77.99
2 h-1	21.48	21.42	91.60	76.05
2 h-2	21.48	21.42	91.92	77.43
2 h-3	21.49	21.42	92.03	79.63
6 h-1	21.48	21.43	91.94	78.05
6 h-2	21.48	21.42	91.62	76.24
6 h-3	21.48	21.42	91.63	76.76
12 h-1	21.42	21.35	91.11	76.89
12 h-2	21.43	21.36	91.56	79.10
12 h-3	21.41	21.33	91.68	79.28
48 h-1	21.47	21.42	92.03	78.5
48 h-2	21.47	21.42	92.25	79.88
48 h-3	21.49	21.3	91.67	77.05

Table S2. Common DEGs involved in signal transduction in virus-infected whiteflies.

Gene ID	Accession No.	Gene Annotation	log2 (Fold change)			
			0 h vs. 2 h	0 h vs. 6 h	0 h vs. 12 h	0 h vs. 48 h
Bta01475	XP_018896986.1	uncharacterized protein LOC109030464	-1.55	-1.67	-1.29	-1.57
Bta01655	XP_018906270.1	rap1 GTPase-activating protein 1 isoform X3	-1.47	-1.21	-1.72	-1.72
Bta02375	XP_018913743.1	uncharacterized protein LOC109041776	-1.18	-1.19	-1.57	-1.95
Bta02580	XP_018905067.1	fatty acid synthase	-1.31	-1.12	-2.05	-2.02
Bta03074	XP_018907085.1	fatty acid synthase	-1.34	-1.20	-1.76	-1.71
Bta04016	XP_018917600.1	ras-like GTP-binding protein RhoL	-1.12	-1.28	-1.07	-1.24
Bta04099	XP_018907804.1	acyl-CoA Delta(11) desaturase-like	-1.28	-1.62	-2.37	-3.34
Bta05107	XP_018895569.1	protein FAM166B-like isoform X2	-1.21	-1.08	-1.15	-1.31
Bta05672	XP_018908790.1	acyl-CoA desaturase-like	-1.92	-1.26	-1.91	-1.48
Bta06172	XP_018896161.1	protein sprint	-1.32	-1.18	-1.33	-1.47
Bta06187	XP_018896237.1	solute carrier family 2, facilitated glucose transporter member 1-like	-1.32	-1.27	-1.35	-1.40
Bta06483	XP_018909499.1	phospholipase A2-like isoform X1	-3.15	-2.25	-2.16	-3.79
Bta07569	XP_018896886.1	fatty acid synthase-like	-1.51	-1.31	-2.05	-2.00
Bta08548	XP_018898043.1	protein decapentaplegic	-1.77	-2.31	-1.48	-1.41
Bta08620	XP_018898020.1	striated muscle-specific serine/threonine-protein kinase-like	-1.26	-1.34	-1.91	-3.07
Bta09047	XP_018916310.1	phosphoenolpyruvate carboxykinase [GTP]-like isoform X2	-1.90	-2.26	-2.51	-2.27
Bta09151	XP_018916388.1	calcium/calmodulin-dependent protein kinase kinase 1 isoform X1	-1.98	-2.10	-2.43	-2.58
Bta09156	XP_018916405.1	insulin-like peptide receptor	-1.95	-1.50	-1.53	-1.34
Bta09525	XP_002430571.1	dual specificity protein phosphatase, putative	-1.48	-1.67	-1.96	-2.03
Bta09906	KPJ01710.1	Nitric oxide synthase-like protein	-1.30	-1.03	-1.21	-1.18
Bta10543	XP_023724614.1	carnitine O-palmitoyltransferase 1, liver isoform isoform X3	-1.17	-1.22	-1.54	-1.64

Bta11166	XP_018912277.1	odium-coupled neutral amino acid transporter 9 homolog isoform X1	-1.23	-1.24	-1.51	-1.61
Bta11403	XP_018900522.1	muscle, skeletal receptor tyrosine protein kinase-like isoform X1	-1.33	-1.13	-1.53	-2.05
Bta11772	XP_022194226.1	sphingomyelin phosphodiesterase	-1.01	-1.36	-1.20	-1.24
Bta12556	XP_018901460.1	senecionine N-oxygenase-like	-1.37	-1.17	-1.33	-1.11
Bta12593	XP_018901501.1	BCL2/adenovirus E1B 19 kDa protein-interacting protein 3 isoform X1	-1.65	-2.00	-2.11	-2.26
Bta15685	XP_018916054.1	ABC transporter G family member 23-like	-1.36	-1.60	-1.87	-1.92

Figures

	0h-1	0h-2	0h-3	2h-1	2h-2	2h-3	6h-1	6h-2	6h-3	12h-1	12h-2	12h-3	48h-1	48h-2	48h-3
0h-1	1.000	0.994	0.998	0.988	0.977	0.977	0.987	0.991	0.977	0.975	0.972	0.984	0.968	0.971	0.970
0h-2	0.994	1.000	0.994	0.984	0.979	0.971	0.982	0.983	0.972	0.973	0.964	0.981	0.953	0.955	0.957
0h-3	0.998	0.994	1.000	0.982	0.970	0.968	0.981	0.987	0.969	0.968	0.963	0.978	0.956	0.959	0.959
2h-1	0.988	0.984	0.982	1.000	0.994	0.996	0.996	0.994	0.995	0.992	0.992	0.997	0.984	0.985	0.987
2h-2	0.977	0.979	0.970	0.994	1.000	0.994	0.990	0.984	0.990	0.992	0.990	0.993	0.974	0.970	0.977
2h-3	0.977	0.971	0.968	0.996	0.994	1.000	0.991	0.987	0.993	0.991	0.994	0.995	0.986	0.984	0.987
6h-1	0.987	0.982	0.981	0.996	0.990	0.991	1.000	0.998	0.995	0.989	0.991	0.996	0.983	0.984	0.985
6h-2	0.991	0.983	0.987	0.994	0.984	0.987	0.998	1.000	0.992	0.986	0.988	0.994	0.983	0.986	0.984
6h-3	0.977	0.972	0.969	0.995	0.990	0.993	0.995	0.992	1.000	0.995	0.998	0.995	0.991	0.991	0.993
12h-1	0.975	0.973	0.968	0.992	0.992	0.991	0.989	0.986	0.995	1.000	0.996	0.994	0.987	0.982	0.989
12h-2	0.972	0.964	0.963	0.992	0.990	0.994	0.991	0.988	0.998	0.996	1.000	0.994	0.993	0.989	0.994
12h-3	0.984	0.981	0.978	0.997	0.993	0.995	0.996	0.994	0.995	0.994	0.994	1.000	0.985	0.985	0.988
48h-1	0.968	0.953	0.956	0.984	0.974	0.986	0.983	0.983	0.991	0.987	0.993	0.985	1.000	0.997	0.998
48h-2	0.971	0.955	0.959	0.985	0.970	0.984	0.984	0.986	0.991	0.982	0.989	0.985	0.997	1.000	0.998
48h-3	0.970	0.957	0.959	0.987	0.977	0.987	0.985	0.984	0.993	0.989	0.994	0.988	0.998	0.998	1.000

1.00
0.95
0.90

Figure S1. Pearson correlation coefficient of gene expression levels among 15 samples.

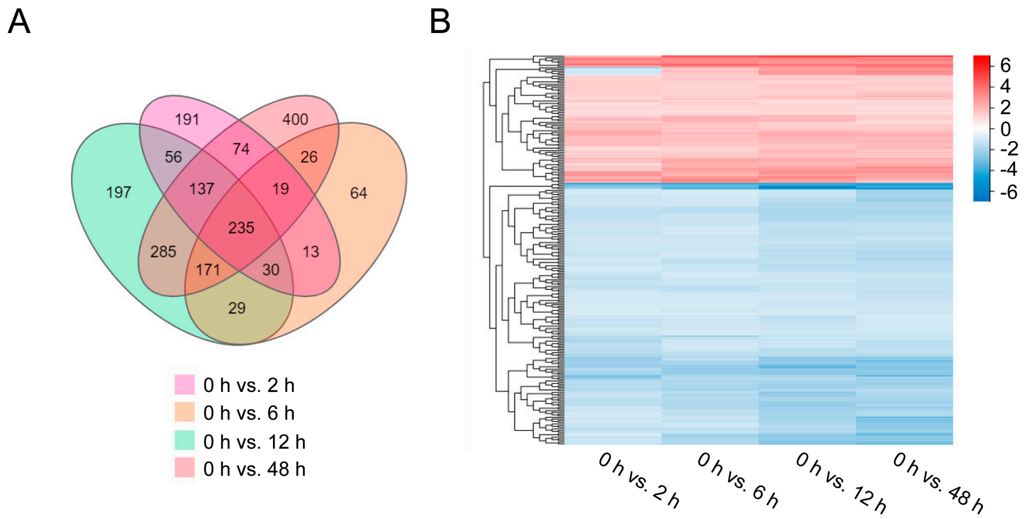

Figure S2. Common DEGs in viruliferous whiteflies. (A) Venn-diagram showing unique and common DEGs in different comparisons of 0 h vs. 2 h, 0 h vs. 6 h, 0 h vs. 12 h and 0 h vs. 48 h. (B) Hierarchical cluster analysis of 235 identified common DEGs.

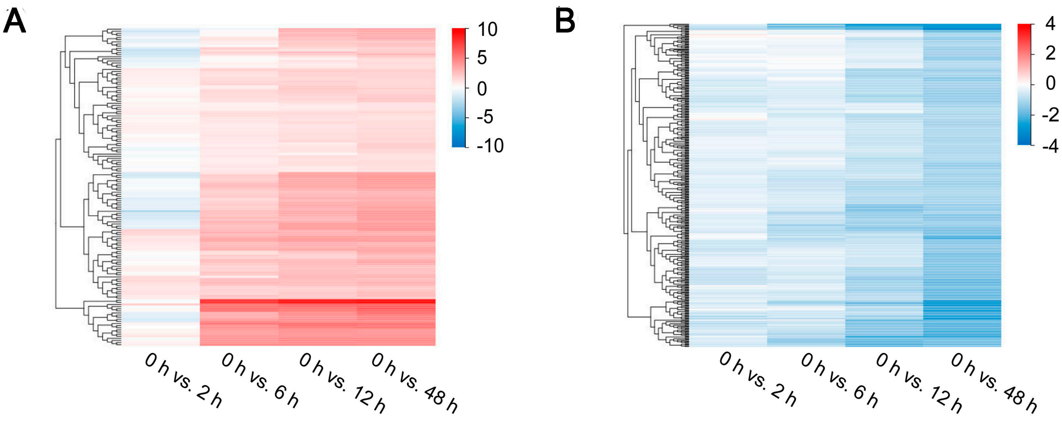

Figure S3. Time-associated gene clusters. For different treatments, genes with similar expression patterns were clustered into different groups. (A) Hierarchical cluster of 127 up-regulated genes. (B) Hierarchical cluster of 355 down-regulated genes.
